# The impact of STING signaling on myeloid cells highlights macrophages as the major player in controlling *Brucella abortus*

**DOI:** 10.3389/fimmu.2025.1727400

**Published:** 2026-01-21

**Authors:** Kívia Q. de Andrade, Nina M. G. P. de Queiroz, Fábio Mambelli, Fábio V. Marinho, Marcelo P. Amaral, Joao Gustavo P. Amarante-Mendes, Glen Barber, Sérgio C. Oliveira

**Affiliations:** 1 Department of Immunology, Institute of Biomedical Sciences, University of São Paulo, São Paulo, SP, Brazil; 2 Department of Biochemistry and Immunology, Institute of Biological Sciences, Federal University of Minas Gerais, Belo Horizonte, MG, Brazil; 3 Center for Innate Immunity and Inflammation, Pelotonia Institute for Immuno-oncology, the James Comprehensive Cancer Center, Department of Surgery, Ohio State University, Columbus, OH, United States; 4 Institut Pasteur de São Paulo, São Paulo, Brazil

**Keywords:** *Brucella abortus*, dendritic cells, inflammasome, macrophages, STING

## Abstract

**Introduction:**

Brucellosis, caused by *Brucella abortus* (*Ba*), is one of the most common zoonoses worldwide. Some components of the bacteria can be recognized by the innate immune system and understanding these mechanisms is necessary for directing future vaccines and therapies against brucellosis. Our group has demonstrated that STING molecule contributes to host protection against *Ba in vivo*. Considering that *Ba* has ability to subvert the immune response, the study of the mechanisms of host cell activation becomes fundamental for the development of measures to control bacterial replication. Currently, there is no data reporting which innate immune cell is important for STING-dependent control of *Ba* infection.

**Methods:**

Therefore, we used conditional knockout animals for STING in dendritic cells (CD11c-SKO) and in macrophages (LysM-SKO). WT, LysM-SKO and CD11c-SKO mice were infected with 1×10_6_ CFU of *Ba* intraperitoneally. In parallel, BMDMs and BMDCs from WT, CD11c-SKO and LysM-SKO mice were infected with *Ba* (MOI 100) for 17 hrs. Next, we evaluated CFU and histology of the liver *in vivo* and LDH, cytokines and pyroptosis *in vitro*.

**Results and Discussion:**

Infected LysM-SKO mice, but not SKO-CD11c, showed an increase in CFU compared to infected WT animals. *In vitro*, the supernatant of infected BMDMs and BMDCs from LysM-SKO and CD11c-SKO animals showed decreased LDH and IL1-β and TNF-α when compared with WT cells. The absence of STING in infected BMDMs decreased the amount of cleaved caspase-1, -11 and GSDMD, evaluated by western blot analysis. The effect of lack of STING was more profound in macrophages than dendritic cells on innate signaling. In both BMDC and BMDM, the absence of STING did not affect NLRP3 expression. Together, these data indicate that STING signaling in macrophages and not in dendritic cells is important to control *Brucella* infection *in vivo* and impacts on inflammasome signaling pathway.

## 1 Introduction

Brucellosis is one of the most common bacterial zoonoses in the world, with a high prevalence in South America. It is caused by Gram-negative, facultative intracellular, non-encapsulated, non-sporulating, and strictly aerobic bacteria belonging to the subphylum α-Proteobacteria (1). Approximately 500,000 new cases are reported per year in humans in endemic areas (2). Three species of the genus Brucella can cause severe brucellosis in humans, including *Brucella abortus* (*B. abortus*), *Brucella melitensis* (*B. melitensis*), and *Brucella suis* (*B. suis*) (3). *B. abortus* infects cattle, causing significant economic losses, and is the most frequent cause of brucellosis worldwide (4). Brucellosis in humans presents several clinical manifestations lasting from days to years (5). It is rarely fatal, but is generally debilitating (6), since *Brucella* has the ability to escape recognition by the host’s immune system (7). Human infection results from the consumption of unpasteurized dairy products, raw meat or through direct contact with tissue from infected animals, such as cattle, sheep, goats and pigs. The bacteria can also reach the individual through the respiratory tract, oral mucosa and conjunctiva (8).

*Brucella* has mechanisms to escape innate immune recognition, affecting the induction of an adequate adaptive immune response (9). Understanding innate cellular immune response, antigen presentation and the induction of the adaptive immune response to *Brucella* infection is important for the development of more effective therapies to control bacterial proliferation.

Despite Brucella’s ability to subvert, some bacterial components can be recognized by the innate immune system, and understanding these mechanisms is necessary for directing future therapies and vaccines against brucellosis. Our research group has demonstrated that *Brucella* genomic DNA is recognized by the AIM2 (absent in melanoma 2) inflammasome (10–12). When recognition by AIM2 occurs, it is followed by the activation of caspase-1, which subsequently cleaves the pro-IL-1β and pro-IL-18 cytokines into their active forms to promote inflammation and inflammatory cell death known as pyroptosis (13). Cell death is important for eliminating infections in cells with the purpose of returning homeostasis (14).

In addition to AIM2 being able to recognize microbial nucleic acids and shape adaptive immunity, the cGAS (cyclic GMP-AMP synthase) protein also detects cytosolic DNA, then generating the second messenger 2’3’-cGAMP that binds to the stimulatory protein of interferon genes (STING), leading to its activation to induce innate immune responses (15–17), through the recruitment and activation of the transcription factor interferon regulatory factor 3 (IRF3) (18, 19). Activated IRF3 then dimerizes and translocate to the nucleus, where it induces the transcription of type I IFN genes. Subsequently, IFN-I signaling can increase the expression of pro-inflammatory cytokines CXCL10 and guanylate-binding proteins (GBPs), which promote the release of bacterial DNA from vacuoles into the cytosol to activate AIM2 and increase IL-1β secretion (10). STING can also induce the activation of the NLRP3 (NOD-, LRR- and pyrin domain-containing protein 3) inflammasome in different ways with subsequent pyroptosis (20, 21) and the cGAS-STING signaling pathway is also involved not only pyroptosis, but also in other types of cell death as apoptosis and necroptosis (22). The STING pathway also induces the activation of NF-κB, which subsequently translocate to the nucleus and induces the expression of several pro-inflammatory genes, as the cytokine TNF-α, creating a microbicidal environment (23). Our group, seeking to understand the participation of STING in infection against *B. abortus*, demonstrated that this molecule is important to host protection against *B. abortus* through the activation of caspase-1, secretion of IL-1β and in the induction of GBP expression (10).

Currently, there is no data reporting which innate immune cell is important for STING-dependent control of *B. abortus* infection, since professional phagocytic cells are the first line of defense in innate immunity against brucellosis. Therefore, we used conditional knockout animals for STING in dendritic cells (CD11c-SKO) and in macrophages (LysM-SKO) as a research model. Studies that evaluate the role of this molecule in macrophages and dendritic cells are of fundamental importance for understanding the immunopathogenesis of brucellosis and may contribute to the establishment of new intervention measures. In conclusion, the results obtained here can serve as a basis for the development of new therapies based on STING activators that can help in the treatment of patients with brucellosis.

## 2 Materials and methods

### 2.1 Mice

Wild-type (WT) C57BL/6 and RIPK3_-/-_ were obtained and maintained in the vivarium of the Department of Immunology at the University of São Paulo (USP). LysM-SKO and CD11c-SKO were obtained from Dr. Glen N. Barber from Ohio State University, Columbus, OH, USA and maintained in the vivarium of the Department of Parasitology at the University of São Paulo (USP). All the animals were used at 6–10 weeks of age. All animal experiments were preapproved by the Animal Use Ethics Committee (CEUA) from University of São Paulo (USP), under protocol number 3271071223.

### 2.2 Mouse bone marrow-derived macrophage and dendritic cell culture

Bone marrow was harvested from mice and differentiated into macrophages (BMDM) by culturing on Petri dishes at 37°C in DMEM (Dulbecco’s Modified Eagle Medium) (Gibco) containing 20% L929 cell supernatant, 10% fetal bovine serum (FBS), 1% penicillin G sodium (100 U/mL), and streptomycin sulfate (100 mg/mL). Cells were fed at day 4 with 10 mL of complete medium (containing the composition described above). One day prior to infection (day 7), cells were plated in DMEM containing 10% FBS. Macrophages were plated at 5×10^5^ cells per well in quadruplicate in 48-well plates and incubated at 37°C.

To generate bone marrow-derived dendritic cell (BMDC), bone marrow cells were differentiated by culturing on Petri dishes in RPMI 1640 (Roswell Park Memorial Institute) containing 10% FBS, 1% penicillin G sodium (100 U/mL), and streptomycin sulfate (100 mg/mL) and 20 ng/mL recombinant GM-CSF (PeproTech 315-03). Cells were fed 5 mL of media on days 3 and 5 and 7. One day prior to infection (day 10), cells were plated in media containing 10% FBS. Dendritic cells were plated at 5×10^5^ cells per well in quadruplicate in 48-well plates and incubated at 37°C.

### 2.3 Flow cytometry analysis

BMDMs (from WT and LysM-SKO) and BMDCs (from WT and CD11c-SKO) were generated as described above and analyzed by flow cytometry. A total of 1x10^6^ cells of each cell type (in triplicate) were centrifuged (450 × g, 10 min, 4°C) and suspended in 25 µL of PBS containing Fixable Viability Stain 450 (1:1000; BD Biosciences) and anti-CD16/CD32 Fc Block (1:100; BD Biosciences) for 10 min at room temperature. Cells were then washed and stained in 50 µL of FACS buffer (PBS supplemented with 0.5% w/v BSA (Bovine Serum Albumin) and 2 mM EDTA; 30 min at 4°C) with the following fluorophore-conjugated monoclonal antibodies for surface markers: anti-CD11b FITC (clone M1/70; BD Bioscience), anti-CD11c PE (clone HL3; BD Bioscience) and anti-F4/80 PE-Cy5 (clone BM8; Biolegend). The appropriate controls were included. Subsequently, cells were washed and fixed/permeabilized with Cytofix/Cytoperm (BD Biosciences). LSRFortessa X-20 (BD Biosciences) flow cytometer was used to collect more than 200,000 events/sample. Data were analyzed using FlowJo vX.0.7 software (Tree Star). Gating strategy and purity assessment are shown in **Supplementary Material**.

### 2.4 Bacterial culture

Bacteria used in this study included *B. abortus* virulent strain S2308 obtained from our laboratory collection. Before being used for cell infection or *in vivo* infection, bacteria were grown in Brucella broth medium (BD Pharmingen, San Diego, CA) for 1 day at 37°C under constant agitation.

### 2.5 Bacterial infections

Mice were infected via intraperitoneal (i.p.) injection of 1×10^6^ CFU of virulent *B. abortus* strain S2308. Animals were kept for two weeks and then killed chemically by ketamine 300mg/kg and xylazine 30mg/kg for CFU counting and pathology. BMDM and BMDC were infected *in vitro* by 17 hrs with virulent *B. abortus* strain S2308 at a multiplicity of infection (MOI) of 100 in DMEM supplemented with 1% FBS (BMDM) or RPMI 1% FBS (BMDC).

### 2.6 *B. abortus* counts in spleens

Five mice from each group of C57BL/6 (WT), LysM-SKO, CD11c-SKO and RIPK3^−/−^ were infected with *B. abortus* S2308 and euthanized as described above. To count residual *Brucella* CFU, the spleen collected from each animal, weighed and processed/macerated in 3 mL of saline solution using two sterile forceps and sterile mesh bags in a sterile petri dish. After the homogenate was serially diluted, and plated in on Brucella Broth agar. After 3 days of incubation at 37°C, the number of CFU was determined as described previously (24).

### 2.7 Histological analysis

Liver fragments were fixed in formaldehyde 10% and subsequently immersed in increasing concentrations of ethanol, before their embedding in paraffin. After processing, histological sections of 5 *μ*m thickness were prepared and stained with hematoxylin and eosin (HE) to evaluate cell microstructures. The stained slides were photographed using an optical microscope connected to a DP70 digital camera system and subsequently evaluated.

### 2.8 Cell death assay

Cells were infected as described, and lactate dehydrogenase (LDH) levels released by dying cells were measured in the supernatant at 17 h after infection. LDH release was quantified using an using the CytoTox 96 LDH-release kit (Promega, Madison, WI), according to the manufacturer’s instructions. Absolute values ​​were obtained by fold change in relation to the uninfected control. Absolute values ​​were expressed in %.

### 2.9 Protein dosage by the bicinchoninic acid assay method and immunoblot analysis

Determination of total protein in cells is necessary for standardization of Western Blot assay. For protein measurement, a 1:10 dilution of the cell homogenate was performed with M-PER (mammalian protein extraction reagent) with NaF, Na_3_VO_4_ and protease inhibitor. The preparation was then vortexed to homogenize. Subsequently, 25 μL of the sample/curve point (BSA) were added in duplicate into the wells of the plate, and 200 μL of the revealing solution were added, waiting for 30 min (protected from light) at a temperature of 37°C. Afterwards, the reading was measured at 562 nm (Pierce BCA Protein Assay Kit (Thermo Fisher Scientific). Protein samples (25 μg) were boiled for 15 min, separated by SDS-PAGE, and transferred to Immobilon-P membranes (Millipore). Samples were then probed with antibodies specific for caspase-1 (Adipogen, San Diego, CA, USA), caspase-11 (Adipogen, San Diego, CA, USA), GSDMD (Abcam, Cambridge, UK) or NLRP3 (Adipogen, San Diego, CA, USA), all at a 1:1000 dilution with TTBS (Tris Buffered Saline with Tween-20) (TBS 1X + 0,1% de tween 20) + 5% BSA, overnight at 4°C. As a loading control, all blots were probed with anti-β-actin (Cell Signaling Technology, Danvers, MA) at a 1:5000 dilution. Detection was performed with HRP-conjugated anti-mouse IgG (Cell Signaling Technology, Danvers, MA) or anti-rabbit IgG (Cell Signaling Technology, Danvers, MA) after an hour and a half of antibody incubation at room temperature. Proteins were visualized using Luminol chemiluminescent HRP substrate (Bio-Rad) in an Amersham™ ImageQuant 800 (Cytiva). Densitometry of caspase-1, caspase-11, GSDMD and NLRP3 was evaluated using ImageJ, normalizing to the corresponding β-actin band for that condition and time point.

### 2.10 Enzyme-linked immunosorbent assays

For *in vitro* tests, cell-free supernatants were used with prior dilution. The levels of cytokines IL-1β (cat. DY401, DuoSet ELISA - R&D Systems), TNF-α (cat. DY410, DuoSet ELISA - R&D Systems) and CXCL10 (cat. DY466, DuoSet ELISA - R&D Systems) were assessed using ELISA with an R&D Systems^®^ kit according to the manufacturer’s instructions.

### 2.11 Quantitative real-time PCR

BMDMs or BMDCs were infected with *B. abortus* in 24-well plates for 17h and homogenized in TRIzol reagent (Invitrogen). Reverse transcription of 1 μg of total RNA was performed in a final volume of 20 μL containing oligo-dT (0.5 μg/μL), dNTP 10 mM, DTT 0.1 M, buffer 5× and reverse-transcriptase (2 U per reaction), using the following cycling parameters: 42°C for 60 min and 70°C for 15 min. Quantitative real-time PCR was conducted in a final volume of 10 μL containing the following: SYBR Green PCR Master Mix (Thermo Fischer Scientific, Waltham, MS, USA), oligo-dT cDNA as the PCR template, and 5 μM of primers. The PCR reaction was performed with QuantStudio 5 Real-Time PCR System (Thermo Fischer Scientific). Primers sequences were as follows: β-actin forward 5′-GGC TGT ATT CCC CTC CAT CG-3′; β-actin reverse 5′-CCA GTT GGT AAC AAT GCC ATG T-3′; IFN-β forward 5′-AGC TCC AAG AAA GGA CGA ACA T-3′; IFN-β reverse 5′-GCC CTG TAG GTG AGG TTG ATC T-3′. Data were analyzed using the threshold cycle (ΔΔ*C*t) method and they were presented as relative expression units after normalization to the house keeping gene *β-actin*.

### 2.12 Statistical analysis

For all variables, normality was assessed using the Kolmogorov–Smirnov test. Parametric variables were evaluated using one-way analysis of variance (ANOVA), followed by *t*-test or Tukey test for comparisons between groups. The Kruskal–Wallis test or Mann–Whitney test was used to evaluate non-parametric variables with corresponding *post hoc* analysis. Results were presented as mean ± standard error (SEM). *p-*values < 0.05 were considered statistically significant. GraphPad^®^Prism version 8.0 for Windows (San Diego, CA, USA) was used.

## 3 Results

### 3.1 STING in macrophages is important for controlling *Brucella abortus* infection *in vivo*

**Figure 1:**
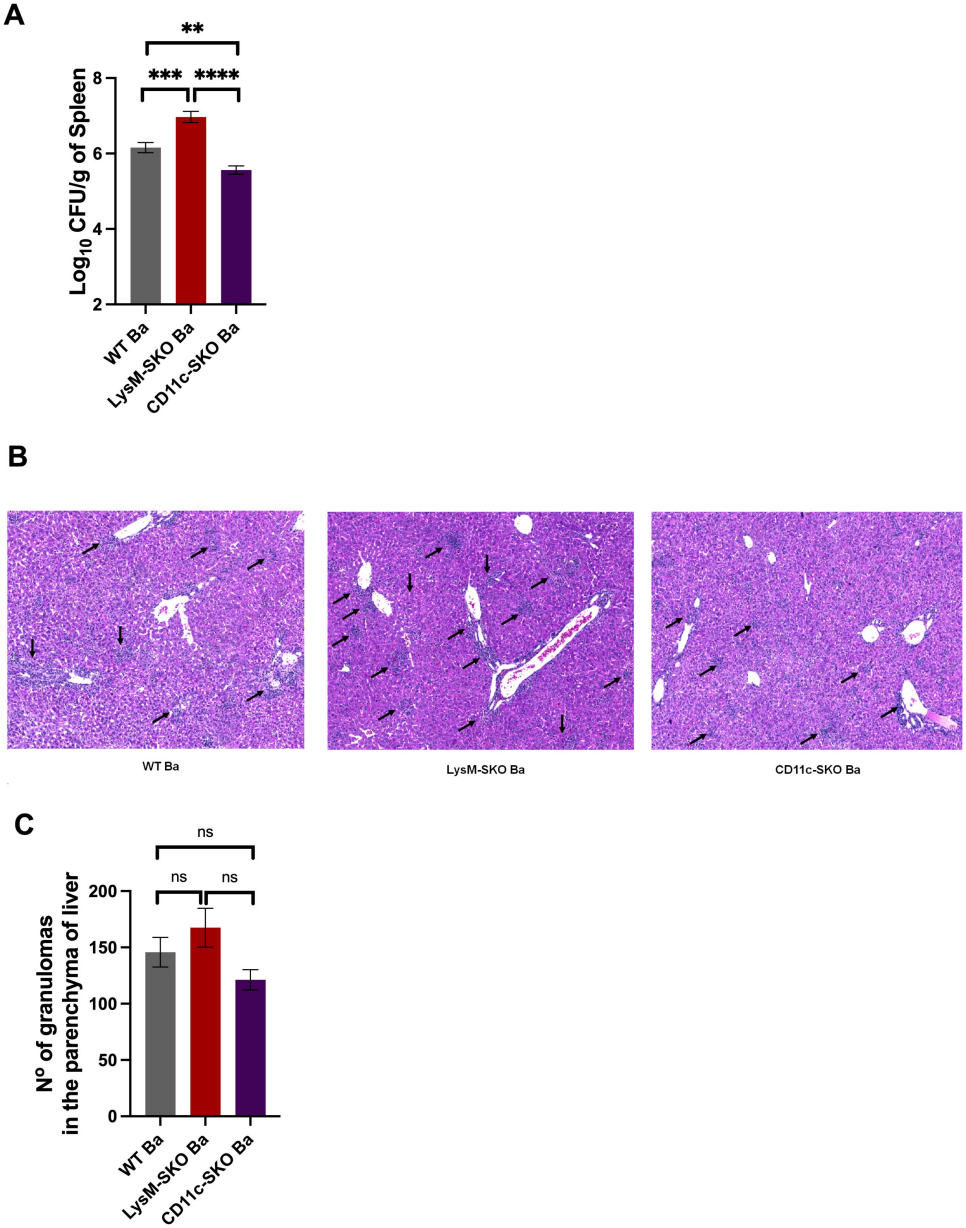
Bacterial load was controlled in LysM-SKO animals. **(A)** CFU levels in the spleen of WT (n=5), LysM-SKO (n=5), and CD11c-SKO (n=5) infected with *Brucella abortus* S2308 (1×10^6^ CFU intraperitoneally) for two weeks. **(B)** Histology of the liver of infected WT, LysM-SKO, and CD11c-SKO animals after euthanasia. Black arrows indicate areas of inflammatory cell infiltrates. **(C)** The resulting slides were evaluated for the number of granulomas in the parenchyma of liver. Data represent the mean ± SEM and were analyzed by one-way ANOVA followed by Tukey’s test. The graphs represent the mean of three independent and reproducible experiments. **p<0.01; ***p<0.001; ****p<0.0001. Ba, *Brucella abortus* S2308; ns, not significant.

Considering that the first line of defense against *Brucella* includes phagocytic cells, such as macrophages and dendritic cells (25, 26), and knowing that STING plays a role in controlling infection *in vivo* (**Supplementary Figure 1**) (10), we decided to determine which of these cells are important for STING signaling during *Brucella* infection. For this purpose, we infected intraperitoneally with 1×10^6^*B. abortus* S2308 CFU C57BL6 (WT) animals and conditional knockout mice for STING in dendritic cells (CD11c-SKO) and in macrophages (LysM-SKO) for 2 weeks. After this period, we observed a significant increase in CFU only in LysM-SKO infected animals compared to infected WT and CD11c-SKO mice (**Figure 1A**). *Brucella* can reach different organs, such as the liver and spleen, through the migration of infected cells to these organs, causing inflammation and permanence within microgranulomas (27). Therefore, we investigated whether the absence of STING in macrophages and/or dendritic cells affected the number of granulomas in the liver parenchyma. After 2 weeks of infection, we observed no statistically significant difference between the WT, LysM-SKO, and CD11c-SKO infected groups (**Figures 1B, C**), even though we detected an increase in absolute numbers in LysM-SKO organ.

Together, these results suggest that STING in macrophages, but not in dendritic cells, is important in the control of *B. abortus in vivo*, and that this phenotype does not appear to affect the number of granulomas in the liver.

### 3.2 Lack of STING in macrophages and dendritic cells affects the production of pro-inflammatory mediators after *Brucella abortus* infection

**Figure 2:**
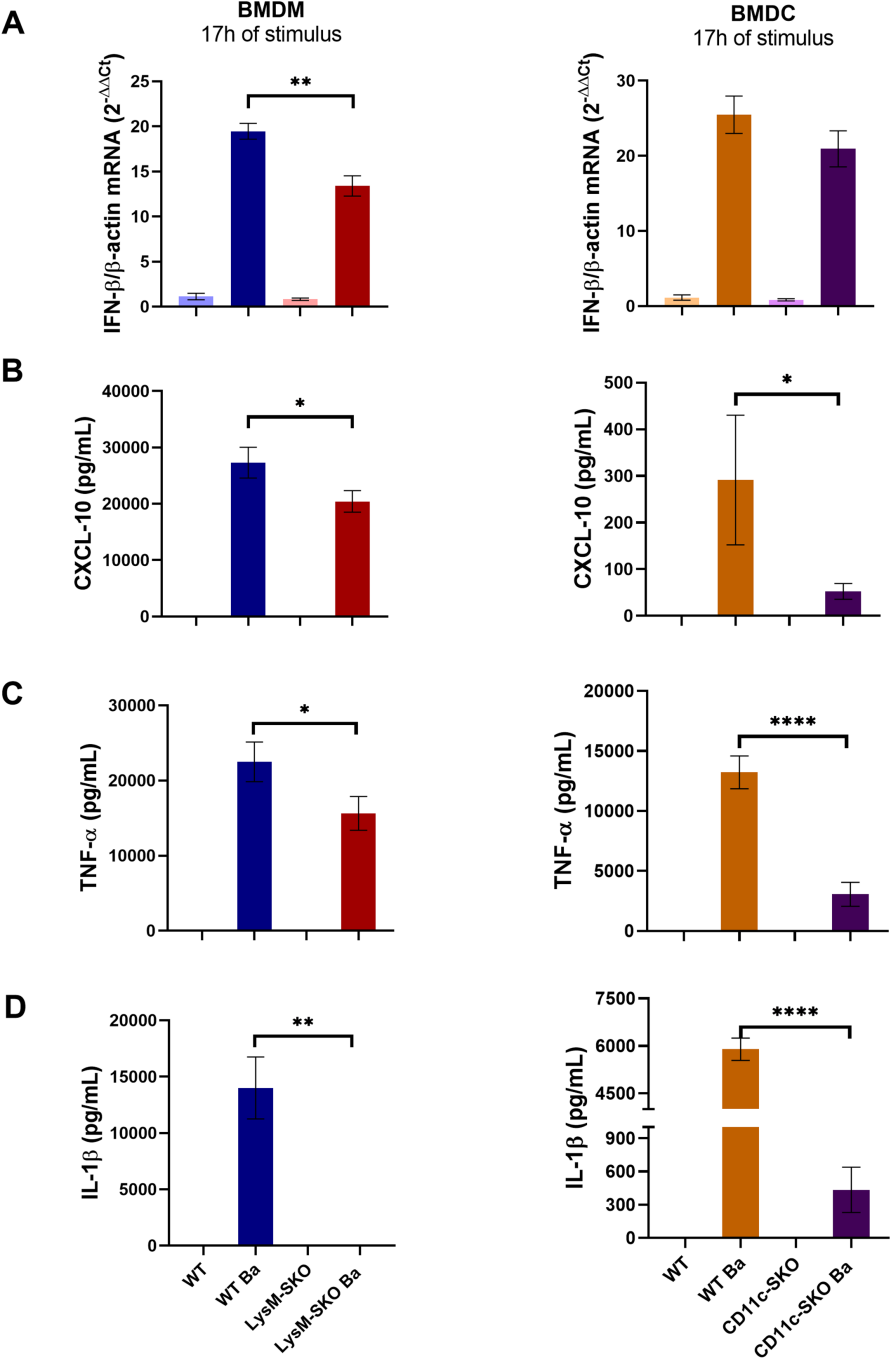
Decrease in pro-inflammatory mediators in infected BMDM and BMDC derived from LysM-SKO and CD11c-SKO animals. **(A)** Total RNA was extracted and qPCR was performed to measure *IFN-β* expression. All qPCR results were relative to *β-actin* mRNA as a normalizer. WT BMDMs unstimulated sample (Mock) was used as control. **(B)** CXCL-10 levels, **(C)** TNF-α levels, **(D)** IL-1β levels in the supernatant of BMDM and BMDC infected with *Brucella abortus* S2308 (MOI 100) for 17 h Data represent the mean ± SEM and were analyzed by one-way ANOVA followed by Tukey’s test. Graphs represent the mean of three independents and reproducible experiments. *p<0.05; **p < 0.01; ****p < 0.0001; Ba: *Brucella abortus* S2308.

To investigate the impact of STING in the induction of pro-inflammatory cytokine secretion in macrophages and dendritic cells, we infect BMDM derived from LysM-SKO and WT mice and BMDC derived from CD11c-SKO and WT mice with *B. abortus* S2308 (MOI 100) for 17 hours. First, we assessed the purity of dendritic cells and macrophages and found approximately 79% double-positive CD11bCD11c cells and 93% double-positive CD11bF4/80 cells (**supplementary Figure 2**). STING induces the expression of type I IFNs via IRF3, a transcription factor required for the induction of *IFN-β* expression (28) and production of the pro-inflammatory cytokine CXCL10 (29). Furthermore, STING induces the activation of the NF-κB pathway, leading to the expression of the pro-inflammatory cytokine TNF-α, and also acts on inflammasome pathways, inducing cell death and IL-1β secretion (21, 30). We observed in the present study that there was a significant decrease in the *IFN-β* expression (**Figure 2A**) in infected BMDM, but not BMDC, derived from LysM-SKO animals compared to infected BMDM derived from WT animals. Furthermore, we found a significant decrease in the secretion of CXCL10 (**Figure 2B**), TNF-α (**Figure 2C**) and IL-1β (**Figure 2D**) in infected BMDM and BMDC derived from LysM-SKO and CD11c-SKO animals compared to infected BMDM and BMDC derived from WT animals.

These results indicate that STING mediates the secretion of the pro-inflammatory cytokine IL-1β in BMDM and BMDC in *B. abortus* infection, while the secretion of CXCL10 and TNF-α in both cells appears to be partially dependent on STING. In macrophages, STING appears to act partially on *IFN-β* expression.

### 3.3 STING appears to be important in the death of BMDM and BMDC infected with *Brucella abortus*

**Figure 3:**
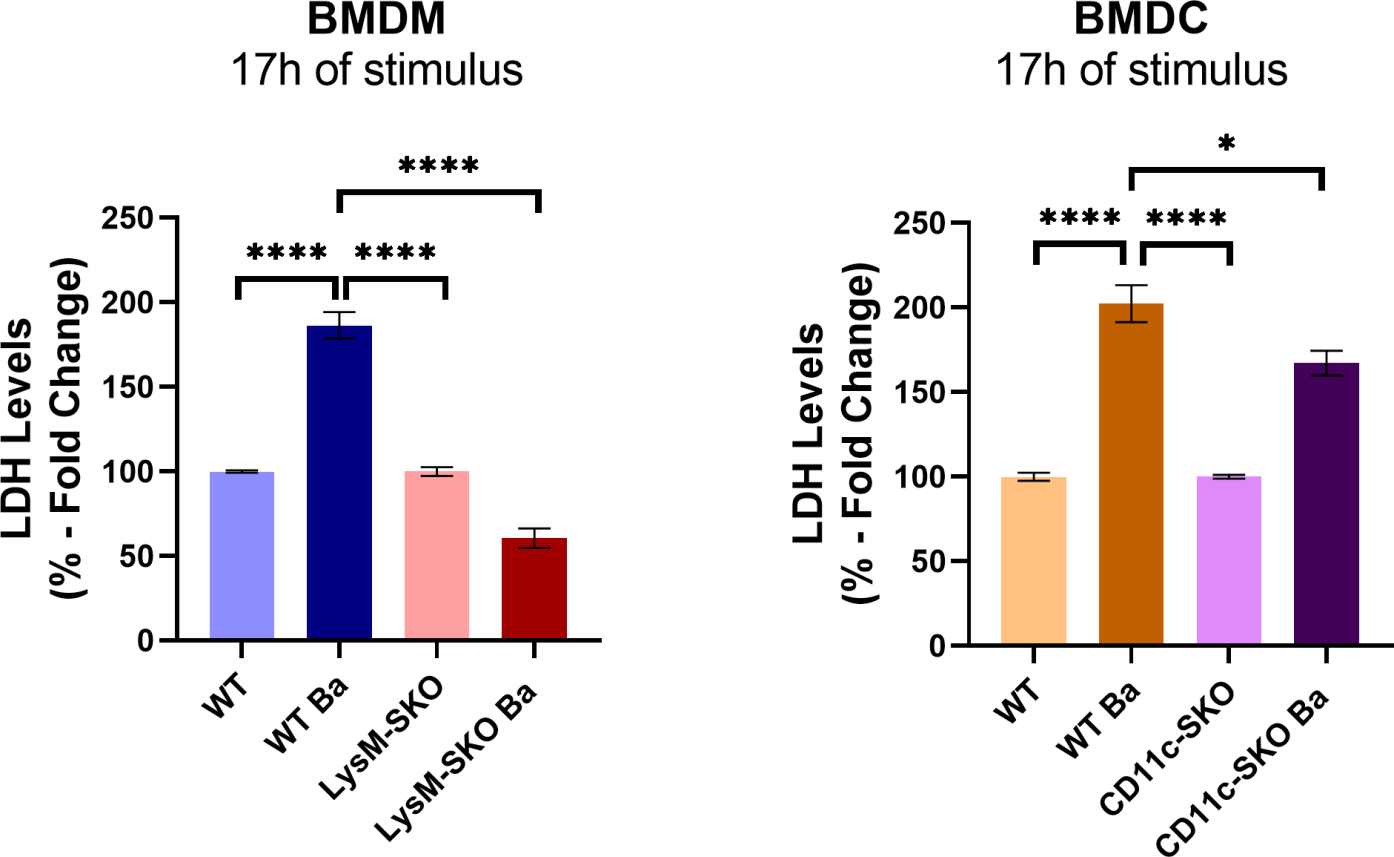
Decreased LDH levels in the supernatant of infected BMDM and BMDC derived from LysM-SKO and CD11_C_-SKO animals. LDH levels in the supernatant of BMDM and BMDC infected with *Brucella abortus* S2308 (MOI 100) for 17 h. Data represent the mean ± SEM and were analyzed by one-way ANOVA followed by Tukey’s test. Graphs represent the mean of three independent and reproducible experiments. *p < 0.05; **** p < 0.0001.

Considering that cell death is an important mechanism in restricting *B. abortus* infection *in vivo* (31), we next evaluated the lactate dehydrogenase (LDH) levels released by dying cells in the supernatant of infected BMDM and BMDC derived from conditional knockout animals for STING and wild-type mice to determine the involvement of STING in cell death during *B. abortus* infection. We infected BMDM derived from LysM-SKO and WT mice and BMDC derived from CD11c-SKO and WT mice with *B. abortus* S2308 (MOI 100) for 17 hours. We observed that there was a significant decrease in LDH in the supernatant of infected BMDM derived from LysM-SKO animals and BMDC from CD11c-SKO animals when compared with infected BMDM and BMDC derived from wild-type mice (**Figure 3**). Additionally, the lack of STING in LysM-SKO macrophages seems to affect more the LDH release than in CD11c-SKO dendritic cells.

These results demonstrate that STING is involved in cell death in BMDM and BMDC during *B. abortus* infection.

### 3.4 STING signaling partially affects pyroptosis in BMDM and BMDC infected with *Brucella abortus*

There is ample evidence demonstrating the interaction between the cGAS-STING signaling pathway and different types of cell death, and the role in infection control and disease pathogenesis (32–34). To investigate the type of cell death that could be involved during *B. abortus* infection, we first infected intraperitoneally WT and RIPK3_-/-_ mice with 1×10^6^ CFU *B. abortus* S2308 for two weeks and subsequently assessed the bacterial load. After the infection period, we observed that there was no significant difference in CFU between WT and RIPK3_-/-_ infected mice (**Supplementary Figure 3**). This finding suggests that necroptosis mediated by RIPK3 does not play a role in controlling *Brucella* infection *in vivo*.

**Figure 4:**
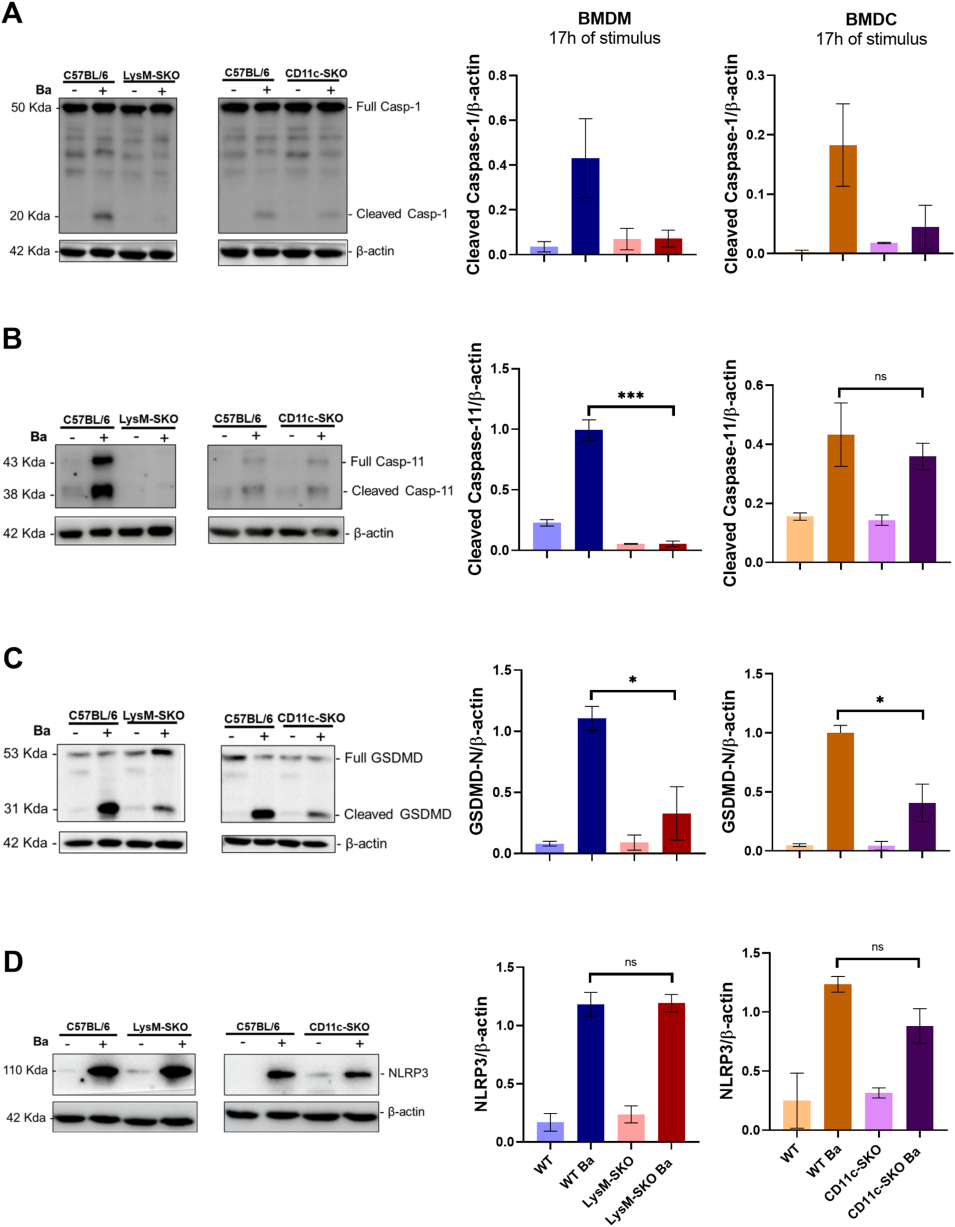
The absence of STING in infected BMDM derived from LysM-SKO animals negatively affects the amount of cleaved caspase-1 and -11 and cleaved GSDMD, but not NLRP3; in infected BMDC derived from CD11c-SKO animals, absence of STING affects only cleaved caspase-1 and cleaved GSDMD. **(A)** cleaved Caspase-1, **(B)** cleaved Caspase-11, **(C)** cleaved GSDMD and **(D)** NLRP3 in the lysate of BMDM and BMDC infected with *Brucella abortus* S2308 (MOI 100) for 17 h, analyzed by western blot. For all targets, the quantification of densitometry is expressed as target relative to the housekeeping protein β-actin. **(A-D)** represent the mean ± SEM and were analyzed using one-way ANOVA, followed by the Tukey’s test. Represents the media of two independents and reproducible experiments. *p < 0.05; ***p < 0.001 Ba, *Brucella abortus* S2308; ns, not significant.

Cerqueira et al. (31) reported that caspase-11 and gasdermin D (GSDMD) (a key components of caspase-initiated pyroptosis) are components of the innate immune response to restrict *B. abortus in vivo*. Therefore, next, we evaluated the role of pyroptosis components involved in inflammasome cell death in infected BMDM and BMDC. We derived cells from WT, LysM-SKO and CD11c-SKO animals and infected them with *B. abortus* S2308 (MOI 100) for 17 hours, seeking to investigate whether STING-mediated signaling could be involved in pyroptosis during bacterial infection. We found in the cell lysate of infected macrophages derived from LysM-SKO animals a reduced amount of cleaved caspase-1, -11 and GSDMD compared to infected cells derived from WT mice (**Figures 4A–C**). While in the lysate of infected BMDC derived from CD11c-SKO animals, we observed less cleaved caspase-1 and cleaved GSDMD, but not of processed caspase-11, in relation to infected dendritic cells derived from WT mice.

There is evidence that activation of the cGAS-STING signaling pathway leads to activation of the NLRP3 inflammasome (35) and also induce NLRP3 expression via IRF3 (20). We then evaluated whether the absence of STING in BMDM and BMDC affects the level of NLRP3 by western blot analysis after 17 hours of infection with *B. abortus* S2308 (MOI 100). We found no significant difference in infected BMDM and BMDC derived from LysM-SKO and CD11c-SKO mice compared to infected BMDM and BMDC derived from WT mice (**Figure 4D**).

Together, these data suggest that STING influences pyroptosis in BMDM and BMDC during *B. abortus* infection.

## 4 Discussion

Brucellosis is a global zoonosis caused by bacteria of the genus *Brucella*, being *Brucella abortus* the most common cause of this disease (4). There is no vaccine for humans, and disease control strategies depend on costly plans (36). Understanding the immunopathogenic mechanisms of brucellosis helps in the development of more effective therapies/strategies. To combat *Brucella*, the first line of defense includes phagocytic actions by innate immune cells, including macrophages and dendritic cells, and activation of PRRs, leading to bacterial processing and antigen presentation (25, 37). Macrophages are one of the main cells that act in the recognition and elimination of pathogens (38), while dendritic cells, despite also acting in phagocytosis, have an essential function as antigen-presenting cells, inducing the activation of adaptive immunity followed by the elimination of the pathogen (39). Macrophages are capable of eliminating a large number of bacteria; however, there is a portion of *Brucella* that escapes the host’s innate immune response, including recognition by PRRs (40–44). Our group reported that *Brucella* genomic DNA is recognized by the AIM2 inflammasome, followed by its activation and subsequent cell death and inflammation (10–12). The cGAS-STING signaling pathway also acts to restrict infections by detecting pathogen DNA, followed by intracellular responses such as type I IFN induction and pro-inflammatory cytokine production (18, 19, 23). Our research team reported in another study that the absence of STING in mice infected with *B. abortus* presented a significant increase in CFU in the spleen compared to cGAS knockout and wild-type C57BL6 animals, demonstrating that STING, but not cGAS, is essential for host defense against *B. abortus in vivo* (10). Therefore, we decided to investigate in this study which myeloid cells are important to STING control of *Brucella*. For this, we used STING conditional knockout animals only in macrophages (LysM-SKO) or dendritic cells (CD11c-SKO). After a 2-weeks of infection with *B. abortus in vivo*, we observed that STING in macrophages, but not in dendritic cells, was important for this control (**Figure 1A**). In dendritic cells, STING, after being activated by cytosolic DNA, increases the production of type I IFN and thus promotes the ability of these cells to present antigens to T cells (45, 46), since type I IFN acts on the maturation of dendritic cells and increases the expression of the major histocompatibility complex (MHC) class I and II (45, 47, 48). While the macrophage is the central element in the cellular immune response to infections with intracellular pathogens such as *Brucella*, it presents several functions such as phagocytic and bactericidal activity (enzymes, acidification, antimicrobial peptides, nutrient deprivation, generation of reactive oxygen and nitrogen species) (49, 50), in addition to producing pro-inflammatory cytokines and chemokines and induce Th1 responses (49, 51, 52). Considering the role of STING in dendritic cells described above, whether all this ultimately leads to successful bacterial control or contributes to pathogenesis depends largely on how the specific bacterium manipulates or is affected by the host’s induced immune responses, particularly type I interferon signaling. In the present study, it was observed that the absence of STING in dendritic cells led to a significant decrease in CFU compared to wild-type animals. Unfortunately, in this study we did not focus in the mechanisms that could explain why the lack of STING in BMDC could lead to increase resistance to infection.

*Brucella* is capable of inducing the formation of granulomas in the spleen and liver of hosts, surrounded by macrophages, since these granulomas can contribute to the persistence of the infection. Typically, an increase in CFU correlates with an increase in granulomas in these organs, demonstrating the severity of the infection, as found in previous studies with *B. abortus* and other bacterial infections such as *Mycobacterium bovis BCG* and *Francisella novicida* (53–57). The literature shows the cell type present in granulomas in some organs, including the liver, during brucellosis. For example, the study of Daggett et al. (58) found, through *in situ* microscopy, an influx of neutrophils and macrophages in the liver during acute infection, and also macrophages, lymphocytes, and neutrophils in chronic infection (58). While in the study by Copin et al. (59), demonstrated that granulomas in the spleen of animals infected with *Brucella melitensis* were mainly composed of CD11b^+^F4/80^+^MHC-II^+^ cells that expressed the iNOS/NOS2 enzyme, and a portion of these cells expressed the CD11c marker with traits similar to those of inflammatory dendritic cells (59). Therefore, we investigated whether the absence of STING in macrophages or dendritic cells *in vivo* affected the number of granulomas in the livers of animals infected for two weeks. After the infection period, we found that the absence of STING in these cells *in vivo* had no significant effect in the number of granulomas in the liver parenchyma (**Figures 1B, C**).

Franco et al. (10) observed by immunofluorescence that *B. abortus* DNA led to STING activation, as well as NF-κB and IRF3 translocation to the nucleus of mouse embryonic fibroblasts (MEFs) derived from wild-type C57BL6 animals (10). The cGAS-STING pathway can induce the activation of the canonical and non-canonical NF-κB pathway, driving the expression of pro-inflammatory cytokines, such as TNF-α (23). This cytokine stimulates macrophages to produce nitric oxide (NO) and reactive oxygen species (ROS), which have a bactericidal function against *B. abortus* (60). In our current study, we identified a significant decrease in TNF-α in BMDM and BMDC derived from LysM-SKO and CD11c-SKO animals compared to BMDM and BMDC derived from wild-type C57BL6 mice (**Figure 2C**), suggesting that *Brucella* also activates NF-κB partially via a STING-dependent pathway.

Additionally, our group also demonstrated that *B*. *abortus* can trigger activation of NLRP3 and AIM2 inflammasomes and caspase-1 and -11 activation leading to IL-1β secretion, a mediator of the immune-protective response against bacteria (11, 12, 31, 61, 62). Using knockout animals for AIM2 or NLRP3 or ASC or caspase-1 or IL-1R or caspase-11, our group demonstrated in a previous study that these animals were more susceptible to infection by *B. abortus* (11, 12, 31), suggesting that the inflammasome pathway is involved in the control of *B. abortus* infection.

The STING signaling is known to induce NLRP3 activation by recruiting this molecule to the endoplasmic reticulum and aiding in the formation of the inflammasome complex (30, 35) furthermore, STING appears to be involved in the removal of NLRP3 polyubiquitination (30), which is important for its activation. STING can also lead to potassium efflux by affecting lysosome permeability and thus leading to NLRP3 inflammasome activation (21). Additionally, STING can also induce the activation of the AIM2 inflammasome, since IFN responses mediated by the cGAS-STING pathway will lead to the release of DNA into the cytosol and thus activate AIM2 inflammasome (63). Both activated inflammasomes induce caspase-1 activation, which activates IL-1β and triggers pyroptosis. Our group also reported in a previous study that the absence of STING in macrophages *in vitro* infected with *B. abortus* or transfected with *B. abortus* DNA, there was a decrease in IL-1β secretion compared to BMDM from wild-type animals (10). Herein, we found a similar result in both BMDM and BMDC. However, in BMDM the decrease in IL-1β in the absence of STING was more robust than the result found in the BMDC supernatant (**Figure 2D**). This difference probably consists of the different ways in which these cells are programmed according to their specific functions and how they act in the inflammatory process (64, 65). Taken together, these results suggest that STING is important adaptor molecule involved in the production of inflammatory cytokines.

Cell death occurs most frequently after infection with intracellular pathogen forming part of an antimicrobial response (66–68). The cGAS-STING pathway regulates different types of cell death, such as apoptosis, necroptosis, pyroptosis, and ferroptosis (22, 33). In our study, we found that the absence of STING in BMDM and BDMC infected with *B. abortus* led to a decrease in LDH, when compared to cells derived from WT animals (**Figure 3**), suggesting that the STING molecule plays a role in cell death during *B. abortus* infection. Although LDH levels in the supernatant of BMDC and BMDM derived from wild-type animals were almost similar, BMDC still showed a greater susceptibility to cell death than BMDM. This may be mainly due to differences in their intrinsic programming related to their distinct functions in the immune system. Macrophages are cells focused on maintaining tissue integrity and removing debris, which requires mechanisms for long-term survival and resistance to cell death. In contrast, dendritic cells are specialized in antigen presentation and migration to lymph nodes, a process that is generally temporary and tightly regulated by cell death pathways to prevent chronic inflammation (69–72).

Therefore, we investigated which type of STING-mediated cell death could be involved in BMDM and BMDC during *B. abortus* infection. TNF-α, through its receptor (TNFR1) on the cell surface, can induce necroptosis, a type of inflammatory cell death, mediated by the sequential activation of RIPK1 (Receptor-interacting serine/threonine-protein kinase 1), RIPK3 (Receptor-Interacting Protein Kinase 3), and MLKL (Mixed lineage kinase domain-like protein) (73–75). In our study, we identified that TNF-α levels were decreased in the supernatant of infected BMDM and BMDC derived from LysM-SKO and CD11c-SKO animals compared to *in vitro* infected cells derived from WT animals (**Figure 2C**). Therefore, we investigated whether RIPK3 knockout mice would have increased CFU after *B. abortus* infection compared to infected WT mice. Since we observed no significant difference in CFU levels of spleen between these groups (**Supplementary Figure 3**), and considering that our previous studies demonstrated the involvement of pyroptosis in macrophages during *B. abortus* infection, we investigated the effect of STING on pyroptosis targets in infected BMDMs and BMDCs. We found that in BMDM derived from LysM-SKO animals there was a decrease in the amount of cleaved caspase-1, -11 and GSDMD (**Figures 4A–C**). Processed GSDMD leads to the formation of pores in the host cell membrane, causing the influx of water with consequent rupture of the cell membrane, thus releasing cytosolic contents, such as LDH, and inducing cell death by pyroptosis (76). In our previous studies, we demonstrated that STING-deficient BMDM infected with *B. abortus* or transfected with *B. abortus* DNA showed a decrease in the amount of activated caspase-1. Furthermore, we found that GSDMD and caspase-11 are required for the induction of pyroptosis in macrophages infected with *B. abortus* (31). In this study, we observed that in infected BMDC derived from CD11c-SKO animals there was a decrease in the amount of cleaved caspase-1 and GSDMD, but not of caspase-11 when compared with infected BMDC derived from wild-type mice (**Figures 4A–C**). Franco et al. (11) also found that the absence of AIM2 in BMDC infected with *B. abortus* resulted in a decrease in the amount of cleaved caspase-1 and IL-1β secretion (11). Other findings presented by their study show that BMDCs infected with *B. abortus* have a higher presence of AIM2 aggregates, suggesting the formation of the inflammasome complex and activation. Furthermore, it was demonstrated that these infected cells presented a colocalization between AIM2 and the bacteria, with AIM2 being more associated with vacuoles containing *Brucella* (BCV) (11). In another study by our group, they also demonstrated the activation of the AIM2 inflammasome in the presence of *B. abortus* DNA, but this time in BMDM, suggesting that the AIM2 inflammasome directly senses *Brucella* DNA intracellularly (10). Regarding pyroptosis, we observed that the impact of STING on LDH levels in BMDM was much greater than in BMDC. This may be due to the importance of both caspase-1 and caspase-11 in the induction of STING-mediated cell death in macrophages, whereas in dendritic cells, caspase-1, rather than caspase-11, appears to be more involved in STING-mediated cell death.

Knowing that STING mediates NLRP3 transcription via IRF3 (20, 77), in the present study we evaluated the impact of STING on the amount of NLRP3 in the cell lysate of BMDM and BMDC derived from WT, LysM-SKO and CD11c-SKO animals after infection with *B. abortus* and found no difference between the infected groups (**Figure 4D**), suggesting that STING is not involved in NLRP3 expression. As a limitation of the current study, we did not investigate all inflammasome receptors that are activated by STING during *Brucella abortus* infection in BMDM and BMDC and whether there is any difference in STING-mediated activation between these cell types which could contribute to understanding the role of the macrophage, and not the dendritic cell, in controlling the bacterial infection.

**Figure 5:**
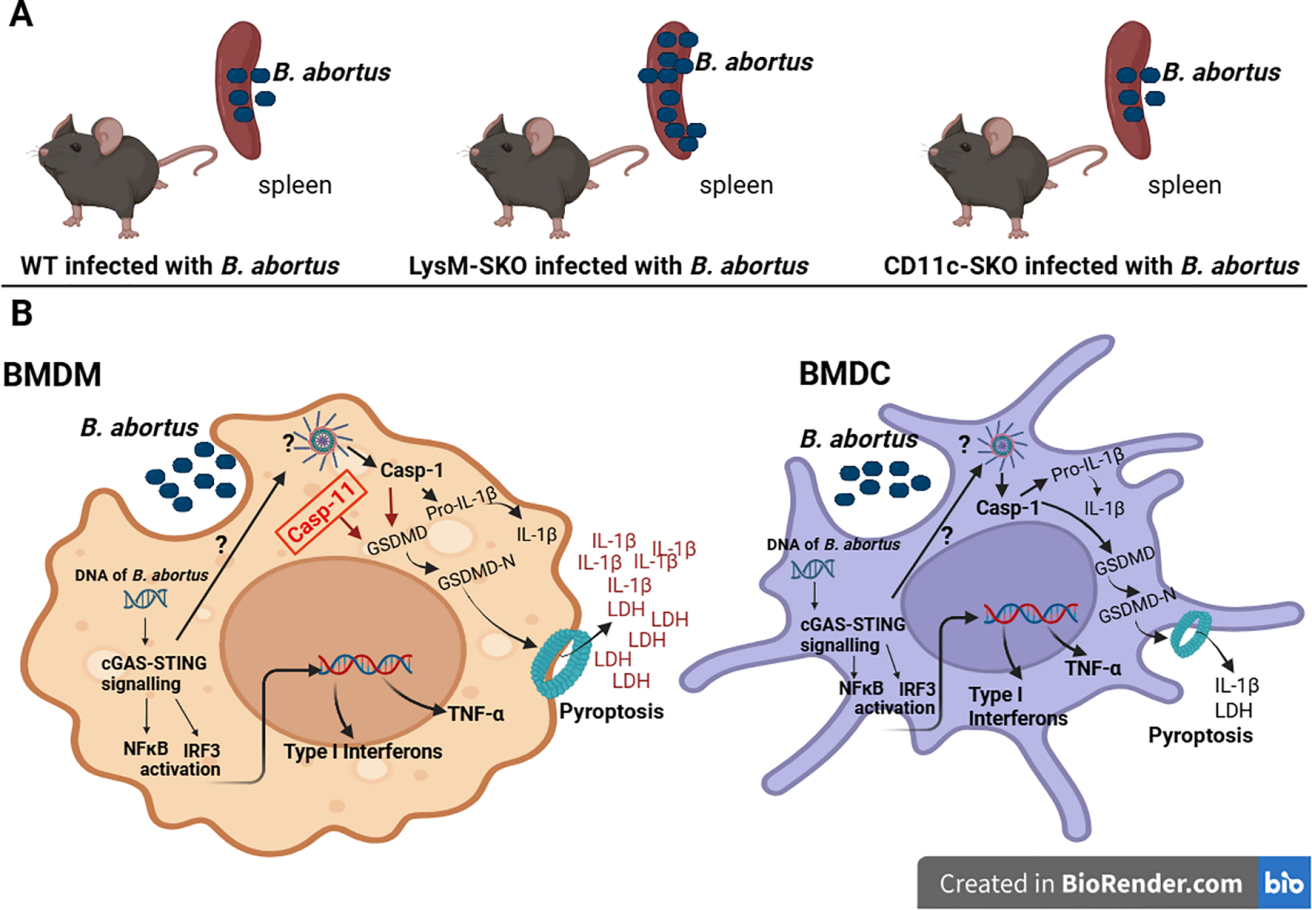
Schematic model proposed for the role of STING in macrophages in controlling *B. abortus* infection. **(A)** STING in macrophages is important for controlling *B.abortus* infection. **(B)** Macrophages and dendritic cells are capable of phagocytosing *B. abortus* bacteria and activating the cGAS-STING signaling pathway, which induces inflammasome activation, synthesis of type I IFNs, and proinflammatory cytokines such as TNF-α. Inflammasome activation leads to caspase-1 activation and GSDMD cleavage, followed by IL-1β secretion and cell death by pyroptosis and LDH release. In macrophages, STING contributes to caspase-11 activation (in red). Both STING-mediated caspase-1 and -11 activation cleave (red arrow) GSDMD, leading to cell death by pyroptosis, an important mechanism for controlling bacterial infection. Thus, in macrophages, there is a more prominent release of IL-1β and LDH than in dendritic cells that may influence host resistance to infection.

These set of results obtained here lead to a model that demonstrate the involvement of STING in inflammasome activation in macrophages and dendritic cells during *B. abortus* infection, represented by caspase-1 activation, IL-1β secretion, caspase-11 and gasdermin D cleavage, and LDH release (**Figure 5**). However, considering the specificity of macrophages in killing bacterial pathogens, our data suggests that STING signaling in macrophages is more important than in dendritic cells to help infection control. Unfortunately, we did not evaluate the possible involvement of other types of cell death mediated by the STING, besides pyroptosis, during *Brucella abortus* infection. These findings contribute to understanding the host-pathogen relationship, contributing to the development of more effective measures for controlling brucellosis targeting the macrophages.

## Data Availability

The raw data supporting the conclusions of this article will be made available by the authors, without undue reservation.
