# Supplementary material for: The impact of STING signaling on myeloid cells highlights macrophages as the major player in controlling Brucella abortus
Source: Front Immunol. 2026 Jan 21;16:1727400. doi: 10.3389/fimmu.2025.1727400 (PMC12868222; doi:10.3389/fimmu.2025.1727400)
Supplement: Supplementary file 1 [file DataSheet1.pdf]

Supplementary Material

A

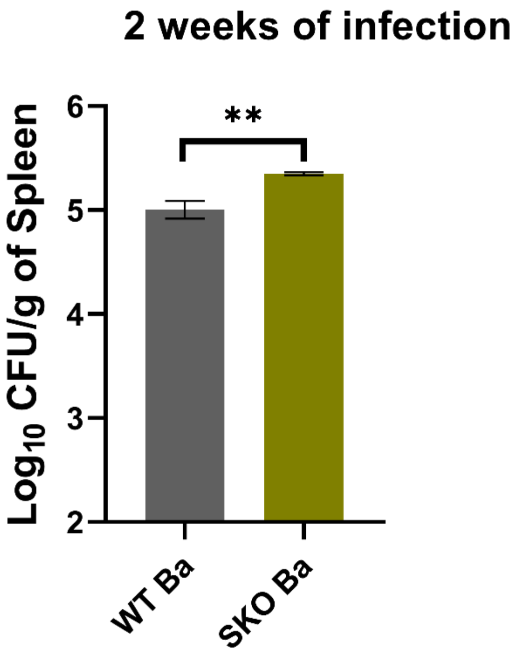

Supplementary Figure 1: Absence of STING affect bacterial load *in vivo*. **(A)** CFU levels in the spleen of WT and STING$^{-/-}$ (SKO) infected with *Brucella abortus* S2308 (1×10^6 CFU intraperitoneally) for two weeks. Data represent the mean±SEM and were analyzed by one-way ANOVA followed by Tukey's test. **$p<0.01$. Ba: *Brucella abortus* S2308.

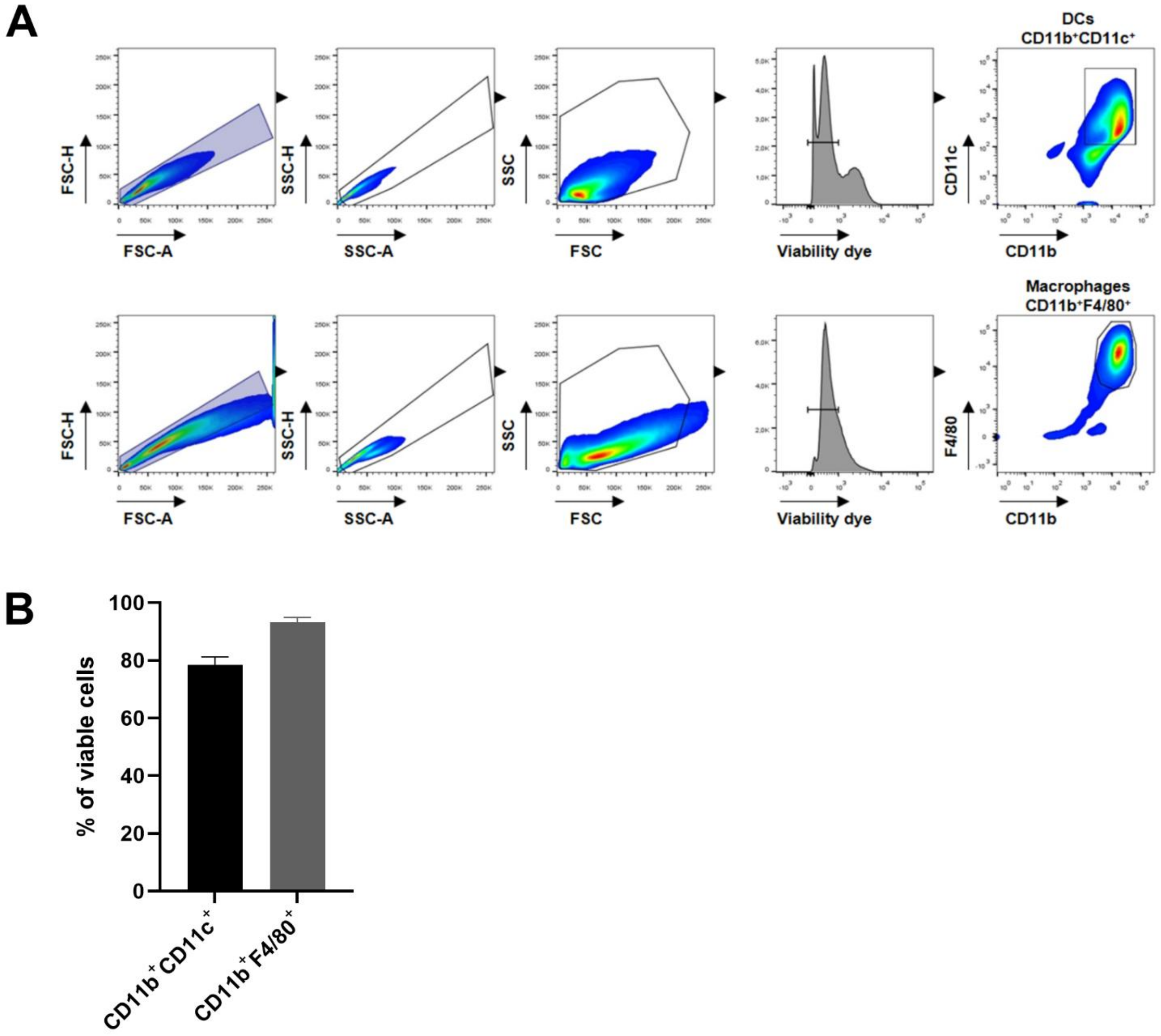

Supplementary Figure 2: Gating strategy for assessment of *in vitro* purity check. **(A)** Single cells were selected and total cells were gated. Next, live cells were discriminated. Upper panel (DCs): cells were gated as $CD11b^{+}CD11c^{+}$ (Dendritic cells). Lower panel (Macrophages): cells were gated as $CD11b^{+}F4/80^{+}$ (Macrophages). **(B)** The percentage of live cells identified as Dendritic Cells or Macrophages is shown.

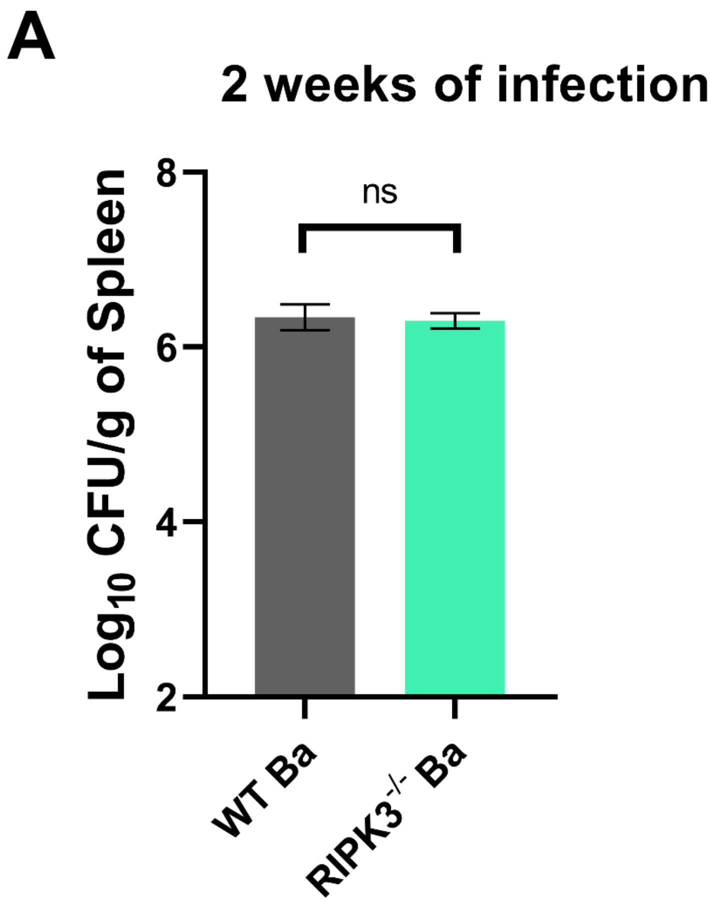

Supplementary Figure 3: Absence of RIPK3 does not affect bacterial load *in vivo*. **(A)** CFU levels in the spleen of WT and RIPK3$^{-/-}$ infected with *Brucella abortus* S2308 (1×10^6 CFU intraperitoneally) for two weeks. Data represent the mean±SEM and were analyzed by one-way ANOVA followed by Tukey's test. Ba: *Brucella abortus* S2308. ns: not significant.

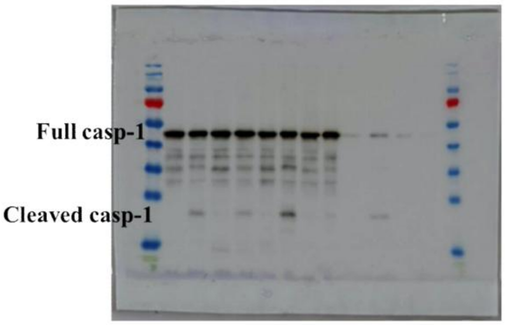

β-actin from caspase-1 membrane

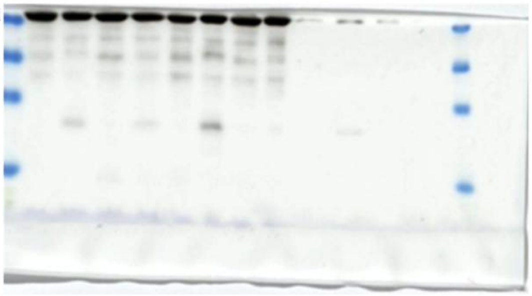

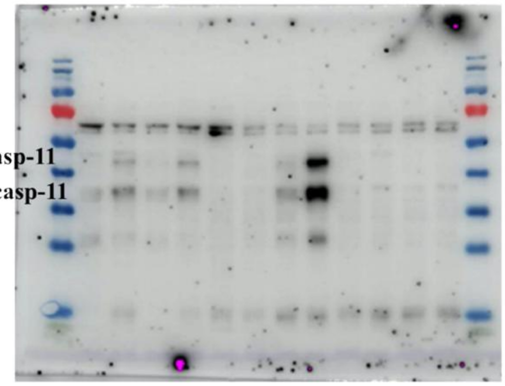

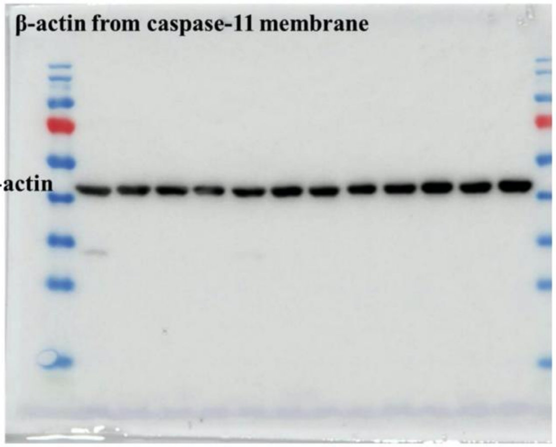

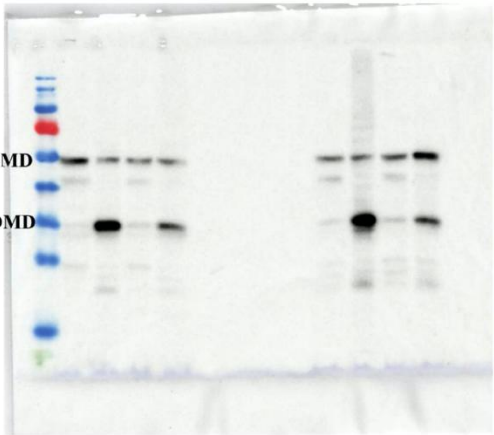

β-actin from GSDMD membrane

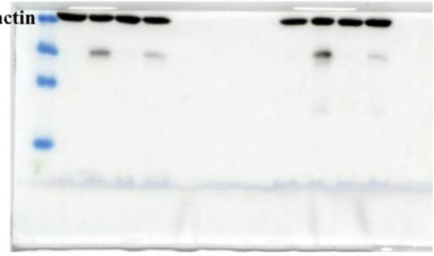

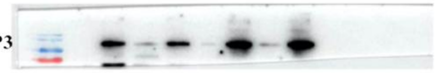

β-actin from NLRP3 membrane

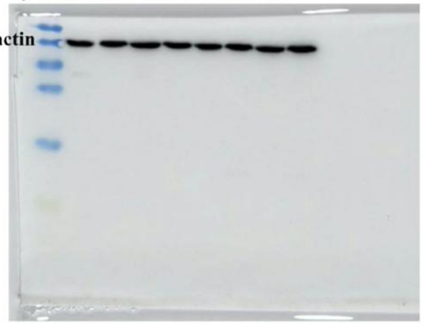

Supplementary Figure 4: Original Western blot membranes (referencing Figure 4) from immunodetection using caspase-1, caspase-11, GSDMD, NLRP3 and β-actin antibodies.
